# Magnetic Resonance Imaging (MRI) Spinal Cord and Canal Measurements in Normal Dogs

**DOI:** 10.1111/ahe.12045

**Published:** 2013-03-15

**Authors:** S Hecht, M M Huerta, R B Reed

**Affiliations:** 1 Department of Small Animal Clinical Sciences, University of Tennessee College of Veterinary Medicine2407 River Drive, Knoxville, TN, 37996, USA; 2 University of Tennessee College of Veterinary Medicine Class of 2013, University of Tennessee College of Veterinary Medicine2407 River Drive, Knoxville, TN, 37996, USA; 3 Department of Biomedical and Diagnostic Sciences, University of Tennessee College of Veterinary Medicine2407 River Drive, Knoxville, TN, 37996, USA

## Abstract

The goal of this study was to establish Magnetic resonance imaging (MRI) reference ranges for spinal measurements in normal dogs. Forty dogs (1–10 kg, 11–20 kg, 21–30 kg, > 30 kg; 10 dogs per category) underwent spinal MRI. Measurements were performed on sagittal T2-W images at the level of the 4th thoracic vertebra (T4), the 9th thoracic vertebra (T9) and the 3rd lumbar vertebra (L3). Spinal canal diameter (mm) ranged from 6.07 ± 0.63 (1–10 kg) to 8.27 ± 1.15 (> 30 kg) at the level of T4; 6.55 ± 0.61 (1–10 kg) to 9.04 ± 1.26 (> 30 kg) at the level of T9; and 6.80 (6.47–7.00; 1–10 kg) to 9.00 (7.90–9.73; > 30 kg) at the level of L3. There were significant differences (*P* < 0.05) in spinal canal diameter between groups. Mean spinal cord diameter (mm) ranged from 4.46 ± 0.51 (11–20 kg) to 4.70 ± 0.35 (1–10 kg) at the level of T4; 4.41 ± 0.50 (> 30 kg) to 4.85 ± 0.57 (1–10 kg) at the level of T9; and 4.52 ± 0.51 (> 30 kg) to 5.14 ± 0.68 (1–10 kg) at the level of L3. There were no significant differences in spinal cord diameter between groups. Spinal cord-to-spinal canal ratio varied significantly, ranging from 0.51 ± 0.08 (> 30 kg at L3) to 0.78 (0.69–0.80; 1–10 kg at T4) (*P* < 0.05). These findings are important when using MRI to evaluate patients with suspected diffuse spinal cord disease.

## Introduction

Imaging of the spine is commonly performed in the diagnostic work-up of cases in small animal practice. While a diagnosis of focal spinal cord lesions such as intervertebral disc herniation, fibrocartilaginous embolism and spinal neoplasia is usually readily accomplished by means of myelography, computed tomography (CT) or magnetic resonance imaging (MRI) (Drost et al., 1996; De Risio et al., 2009; Hecht et al., 2009; Nakamoto et al., 2009; Beltran et al., 2012; Mankin et al., 2012), imaging diagnosis of diffuse spinal cord diseases such as diffuse myelitis or degenerative disorders remains elusive (Gnirs, 2006; Tamura et al., 2007; Polizopoulou et al., 2008). A myelographic finding of a diffusely swollen cord has been reported in dogs with ascending myelomalacia (Lu et al., 2002). In dogs afflicted with degenerative myelopathy, myelography combined with computed tomography may demonstrate a smaller cord than is seen in normal dogs (Jones et al., 2005). However, anatomical differences between dogs of different sizes and breeds may render these findings equivocal. In recent years, MRI has become the gold standard for most applications in veterinary neuroradiology (Hecht and Adams, 2010; Dennis, 2011) necessitating definition of normal spinal dimensions in dogs. The goal of this study was to establish MRI reference ranges for spinal cord and spinal canal measurements in normal dogs. The hypotheses were that an increase in spinal cord and spinal canal diameter would be noted with increasing weight, and that the spinal cord-to-spinal canal ratio would remain constant between different weight groups.

## Materials and Methods

### Preliminary study

A fresh cadaver of a young adult mixed breed dog weighing approximately 15 kg euthanized for reasons unrelated to spinal disease was obtained from a local animal shelter to determine the optimal MRI sequence for accurate spinal measurements. The dog was positioned in dorsal recumbency. Sagittal T1-weighted (T1-W; TR 301 ms, TE 14 ms) and T2-weighted (T2-W; TR 2270 ms, TE 126 ms) images were obtained using a 1T MRI system (Magnetom Harmony™; Siemens Medical Solutions, Malvern, PA, USA) and were sent to a picture archival and communications system (Sectra PACS; Sectra North America Inc., Shelton, CT, USA) for further evaluation. MRI measurements were made on mid-sagittal MRI images for the vertebral bodies Th11-L4. Mid-sagittal slice location was assured by placing a single initial slice on midline using the spinous process and ventral ridge denoting the median part of the vertebral body on sagittal and dorsal localizer images, and subsequently adding an even number of parallel paramedian slices to the left and right of the initial slice. The spinous processes were visible on the resultant mid-sagittal image allowing its unequivocal identification. Images were magnified if needed to assure best visibility of anatomical boundaries. Dorsoventral spinal canal and spinal cord diameter were measured perpendicular to the spinal cord at the widest point of the spinal canal over the respective vertebral body. The evaluation was performed by a veterinary student (M.M.H) in collaboration with a board certified radiologist (S.H.) experienced in MR interpretation. Each measurement was made 3 times, and the mean of these measurements was calculated. Subsequently, the cadaver was frozen and cut in sagittal section using a bandsaw. Measurements of spinal canal and spinal cord diameter were performed at the same levels and following the same principles as the MRI measurements. Pearson product–moment correlation revealed better correlation of T2-W images than of T1-W images with anatomical measurements (*r* = 0.458 versus *r* = 0.400 for spinal cord measurements; *r* = 0.699 versus *r* = 0.471 for spinal canal measurements). Based on this preliminary study, T2-W images were used for the remainder of the study.

### Sample population

**Figure 1:**
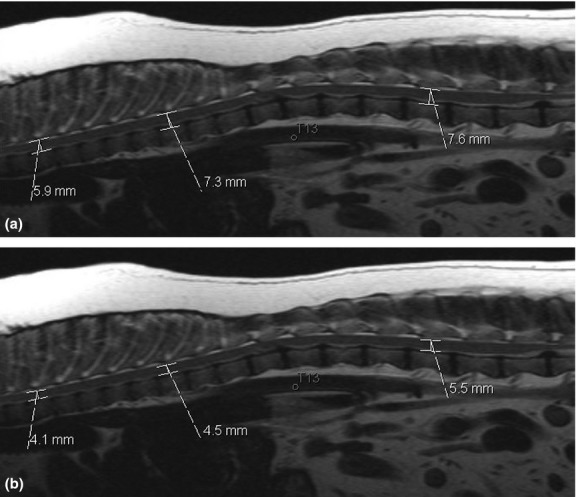
Spinal canal (a) and spinal cord (b) measurements at the level of the vertebral bodies T4, T9 and L3 in a dog weighing 11 kg. Note intervertebral disc herniations at T12/13 and L5/6, distant from sites of measurements.

**Figure 2:**
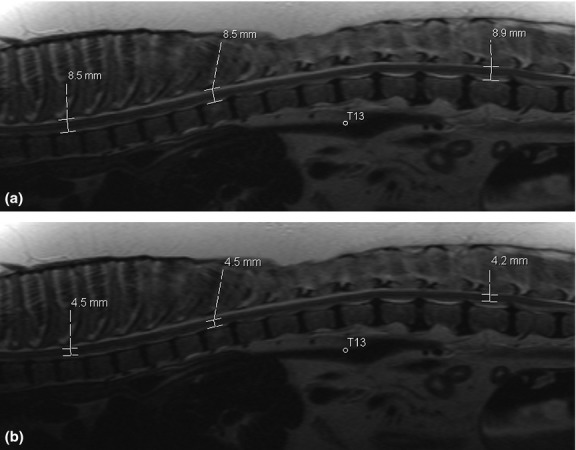
Spinal canal (a) and spinal cord (b) measurements at the level of the vertebral bodies T4, T9 and L3 in a dog weighing 42 kg. Note incidental mild multifocal intervertebral disc bulges.

The MRI database of the University of Tennessee College of Veterinary Medicine was searched for dogs in which an MRI examination of the thoracolumbar spine had been performed and for which well-positioned mid-sagittal images were available. All dogs were scanned using the same MR system as described for the cadaver above. Dogs with a presumptive or proven diagnosis of spinal disease potentially resulting in a generalized increase or decrease in spinal cord diameter (e.g. myelitis or degenerative myelopathy) were excluded from the study. Animals were subdivided into four categories according to weight (1–10 kg, 11–20 kg, 21–30 kg, > 30 kg). Ten suitable dogs were identified per category. Mean age was 5.6 ± 2.4 years. Twenty different breeds were represented. There were 21 ovariohysterectomized females, three sexually intact females, 14 castrated males and two sexually intact males. Spinal measurements were performed on mid-sagittal T2-W images (TR 1900–2890 ms, TE 126 ms, slice thickness 3 mm) at the level of T4, T9 and L3 as described above (Figs 1 and 2).

### Statistical evaluation

Statistical analysis was performed using statistical software (Microsoft Excel; Microsoft Corp, Redmond, WA, USA; SigmaStat® 3.1; Systat Software Inc, Richmond, CA, USA). Data are presented as mean (± SD) for normally distributed data and median (range) for non-normally distributed data. One-way anova or Kruskal–Wallis anova on ranks were used for comparison between groups. A *P* value of < 0.05 was considered significant.

## Results

**Figure 3:**
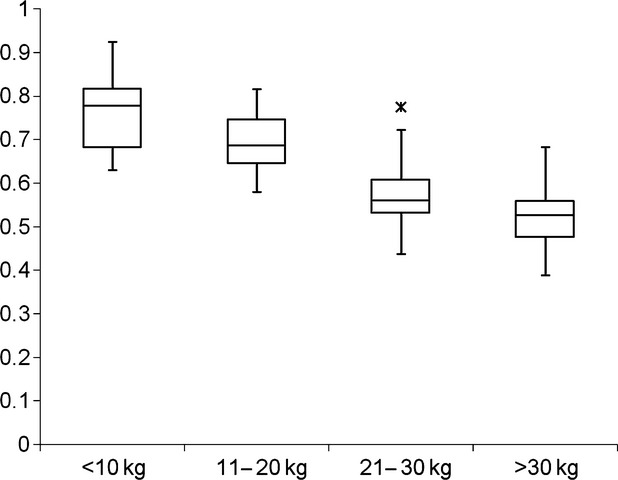
Schematic representation of the spinal cord-to-canal ratio for dogs of different weight groups. All measurements (T4, T9 and L3) were included to produce this graph. Each box represents the inter-quartile range from the 25th to the 75th percentiles. The horizontal bar through the box is the median. The ‘whiskers’ represent the main body of data. Outlying data points are represented by asterisks. There is a significant decrease in spinal cord-to-spinal canal diameter in the thoracolumbar spine of dogs with increasing weight.

Data are summarized in Tables 1–3. Mean/median spinal canal diameter (mm) ranged from 6.07 ± 0.63 (1–10 kg) to 8.27 ± 1.15 (> 30 kg) at the level of T4; 6.55 ± 0.61 (1–10 kg) to 9.04 ± 1.26 (> 30 kg) at the level at T9; and 6.80 (6.47–7.00; 1–10 kg) to 9.00 (7.90–9.73; > 30 kg) at the level of L3. There were significant differences in spinal canal measurements between groups (*P* < 0.05). Mean spinal cord diameter (mm) ranged from 4.46 ± 0.51(11–20 kg) to 4.70 ± 0.35 (1–10 kg) at the level of T4; 4.41 ± 0.50 (> 30 kg) to 4.85 ± 0.57 (1–10 kg) at the level of T9; and 4.52 ± 0.51 (> 30 kg) to 5.14 ± 0.68 (1–10 kg) at the level of L3. There were no significant differences in spinal cord measurements between groups. Spinal cord-to-spinal canal ratio varied significantly between different weight groups, ranging from 0.51 ± 0.08 (> 30 kg at L3) to 0.78 (0.69–0.80; 1–10 kg at T4) (*P* < 0.05) (Fig. 3).

**Table 1:** Results of spinal canal, spinal cord and spinal cord-to-canal ratio at the level of T4

	Canal (mm)	Cord (mm)	Cord/canal ratio
1–10 kg	6.07 ± 0.63**†	4.70 ± 0.35†	0.78 (0.69–0.80)**†
11–20 kg	6.34 ± 0.70**†	4.46 ± 0.51†	0.67 (0.67–0.76)**†
21–30 kg	7.87 ± 0.72**‡	4.50 ± 0.26†	0.56 (0.53–0.57)**‡
> 30 kg	8.27 ± 1.15**‡	4.60 ± 0.60†	0.55 (0.53–0.59)**‡

* Statistically significant difference between groups (*P* < 0.05).

†‡ No significant difference between groups.

**Table 2:** Results of spinal canal, spinal cord and spinal cord-to-canal ratio at the level of T9

	Canal (mm)	Cord (mm)	Cord/canal ratio
1–10 kg	6.55 ± 0.61**†	4.85 ± 0.57†	0.74 ± 0.09**†
11–20 kg	6.74 ± 0.70*§†	4.66 ± 0.44†	0.74 ± 0.06**†
21–30 kg	7.80 ± 1.46*‡§	4.51 ± 0.32†	0.60 ± 0.11***
> 30 kg	9.04 ± 1.26**‡	4.41 ± 0.50†	0.49 ± 0.05***

* Statistically significant difference between groups (*P* < 0.05).

†‡§ No significant difference between groups.

**Table 3:** Results of spinal canal, spinal cord and spinal cord-to-canal ratio at the level of L3

	Canal (mm)	Cord (mm)	Cord/canal ratio
1–10 kg	6.80 (6.47–7.00)**†	5.14 ± 0.68†	0.76 ± 0.09**†
11–20 kg	7.08 (6.37–7.47)*†§	4.71 ± 0.32†	0.68 ± 0.08**†
21–30 kg	8.17 (8.07–9.00)*§‡	4.73 ± 0.51†	0.58 ± 0.08**‡
> 30 kg	9.00 (7.90–9.73)**‡	4.52 ± 0.51†	0.51 ± 0.08**‡

* Statistically significant difference between groups (*P* < 0.05).

†‡§ No significant difference between groups.

## Discussion/Conclusions

This study was performed to establish normal reference ranges for spinal cord, spinal canal and spinal cord-to-canal diameter based on MRI measurements in normal dogs. The location of measurements (vertebral bodies T4, T9 and L3) was chosen based on the following considerations: (1) In medium and large breed dogs, the conus medullaris ends approximately at the level of the L6-7 intervertebral disc, while small dogs have relatively longer spinal cords (Fletcher, 1993; Scrivani, 2000). (2) The cervical and lumbar intumescences which give rise to the nerves contributing to the brachial and lumbosacral plexus are naturally occurring areas of increased spinal cord diameter. These cervical and lumbosacral enlargements lie at the sixth and seventh cervical and fourth and fifth lumbar vertebrae, respectively (Dyce et al., 2002). To avoid these natural enlargements, the cranial most measurement was performed caudal to the cervical intumescence, and the caudal most measurement was performed cranial to the expected location of the lumbar intumescence. While a significant increase in spinal canal diameter was noted with increasing weight as hypothesized, no significant differences were noted in spinal cord diameter between weight groups, and the spinal cord-to-spinal canal ratio was significantly smaller in larger dogs. These findings are in agreement with a previous study which found a higher ratio of spinal cord-to-vertebral canal height in Dachshunds than in German Shepherd Dogs based on myelographic measurements (Morgan et al., 1987). A higher spinal cord-to-vertebral canal ratio has also been reported for the cervical spine in smaller compared with larger dogs based on myelographic findings (Fourie and Kirberger, 1999). From a clinical perspective, it could be speculated that presence of extradural material in the spinal canal (e.g. secondary to intervertebral disc herniation) might result in earlier/more dramatic clinical signs in smaller than larger dogs as there is less room to accommodate extraneous material in addition to spinal cord parenchyma.

There are several limitations to this study. (1) Measurements were limited to the thoracolumbar spine, and cervical measurements were not performed. However, as most dogs with degenerative myelopathy present with a T3-L3 neurolocalization (Coates and Wininger, 2010) rather than signs referable to the cervical spine, data presented in this study may be helpful in the diagnostic work-up of these patients. Normal cervical spinal measurements based on MRI data could be established in a future study. (2) Only 40 dogs were included in the study. While this is a fairly small sample size, it is comparable to previous similar studies (Fourie and Kirberger, 1999), and it was adequate to produce statistically significant results. (3) Another limitation is the grouping of dogs according to weight, as inclusion of overweight or cachectic dogs may have skewed our results. This is a common problem when investigating client owned animals (Fourie and Kirberger, 1999) and could be addressed by taking into account the patients' body condition in future studies. (4) Dogs were excluded if there was suspicion or proof of spinal disease resulting in generalized decrease or increase in spinal cord size. Special care was taken to exclude large breed dogs with clinical signs compatible with degenerative myelopathy, dogs with MRI findings suggesting an intramedullary lesion and dogs with abnormalities detected on cerebrospinal fluid analysis. Necropsy was not performed in any of these dogs, however, which would have been the gold standard to definitively rule out diffuse spinal cord disease. While this is a limitation of the study, it is a recognized and common problem in studies utilizing client owned animals (Morgan et al., 1987; Fourie and Kirberger, 1999; Hecht et al., 2009; Mankin et al., 2012). (5) Measurements were performed on sagittal rather than transverse images. The mid-sagittal image was chosen based on visualization of the spinous process to assure measurements were performed at the maximum diameter of spinal canal and spinal cord, respectively. It is conceivable that the spinal cord could have been located off midline within the vertebral canal in some dogs which could have negatively affected accuracy of measurements. However, a complete MRI study was performed in all dogs, and dogs with focal lesions at the measurement sites were excluded. As sagittal MR images of the spine are routinely acquired prior to planning transverse slices (Dennis, 2011; Guillem Gallach et al., 2011), this is most likely the slice orientation which would be used to evaluate spinal cord and spinal canal diameter in clinical patients. (6) Only T1-W and T2-W sequences were evaluated in the frame of the preliminary cadaveric study. While the use of advanced sequences such as half-Fourier-acquisition single-shot turbo spin-echo (Pease et al., 2006; Mankin et al., 2012), short tau inversion recovery (Dennis, 2011) and post-contrast T1-W with chemical fat saturation (Freeman et al., 2012) has been advocated for spinal imaging in small animals, these are not a routine part of the examination at many institutions. Future studies could be performed to determine agreement between different MRI sequences when performing spinal measurements.

In conclusion, we found a significant decrease in spinal cord-to-spinal canal diameter in the thoracolumbar spine of dogs with increasing weight. These measurements can serve as a baseline when evaluating clinical patients with suspected diffuse spinal cord disease.
